# Sylvatic vector-borne pathogens including *Cytauxzoon europaeus* in the European wildcat (*Felis silvestris*) from southwestern Germany

**DOI:** 10.1186/s13071-024-06428-w

**Published:** 2024-08-24

**Authors:** Anna Obiegala, Luisa Fischer, Sara Weilage, Nina Król, Katharina M. Westhoff, Saskia Nemitz, Michael Lierz, Johannes Lang, Martin Pfeffer, Zaida Renteria-Solís

**Affiliations:** 1 Institute of Animal Hygiene and Veterinary Public Health, University of Leipzig, Leipzig, Germany; 2 Wildlife Research Institute, State Agency for Nature, Environment and Consumer Protection North Rhine-Westphalia, Bonn, Germany; 3 Working Group for Wildlife Biology at Justus, Liebig University Giessen E.V., Giessen, Germany; 4 Department of Veterinary and Animal Sciences, University of Copenhagen, Frederiksberg, Denmark; 5 Clinical Center for Emerging and Vector-Borne Infections, Odense University Hospital, Odense, Denmark; 6 Clinic for Birds, Reptiles, Amphibians and Fish, Working Group for Wildlife Research, Justus Liebig University Giessen, Giessen, Germany; 7 Institute of Parasitology, Centre for Infection Medicine, Faculty of Veterinary Medicine, University of Leipzig, Leipzig, Germany

**Keywords:** Tick-borne pathogens, *Cytauxzoon europaeus*, *Rickettsia helvetica*, *Anaplasma phagocytophilum*, *Bartonella taylorii*, Piroplasmida, Europe

## Abstract

**Background:**

European wildcats (*Felis silvestris*) are widely distributed in Europe and a strictly protected species in Germany. Lately, anthropogenic protective efforts lead to increasing numbers of wildcats in southwestern Germany. Moreover, in recent years the numbers of domestic cats are increasing. Thus, the contact between domestic and wildcats may lead to the spread of zoonotic pathogens in both animal species. As data on vector-borne pathogens (VBPs) in wildcats from Germany are limited to date, the objective of this study was to investigate the presence and current distribution of VBPs in wildcats from southwestern Germany.

**Methods:**

Skin and spleen samples from 117 European wildcats, originating from a regional carcass-monitoring program in southwestern Germany, were examined by real-time and conventional polymerase chain reaction (PCR) for the presence of *Anaplasma phagocytophilum*, *Neoehrlichia mikurensis*, *Rickettsia* spp., *Bartonella* spp., and Piroplasmida.

**Results:**

In total, 6.8% (*n* = 8) of the wildcats were *Rickettsia*-positive, specified as *R*. *helvetica*. Three wildcats were positive for *A*. *phagocytophilum* (2.6%), one for *Bartonella* spp., namely *B*. *taylorii* (0.8%), and 84 for *Cytauxzoon* spp. (71.8%). Out of these 84 samples, 23 were further sequenced revealing very high identity levels (99.84–100%) to *C*. *europaeus*, which is considered to be pathogenic for domestic cats. All wildcats were negative for the presence of *N*. *mikurensis* DNA.

**Conclusions:**

European wildcats in southwestern Germany are hosting several VBPs. With the exception of *Cytauxzoon* spp., low prevalence rates of most examined pathogens suggest that wildcats are primarily incidental hosts for sylvatic pathogens associated with rodents, in contrast to domestic cats. However, the high prevalence of the cat-associated pathogen *C*. *europaeus* suggests that wildcats in southwestern Germany may serve as reservoirs for this pathogen.

**Graphical Abstract:**

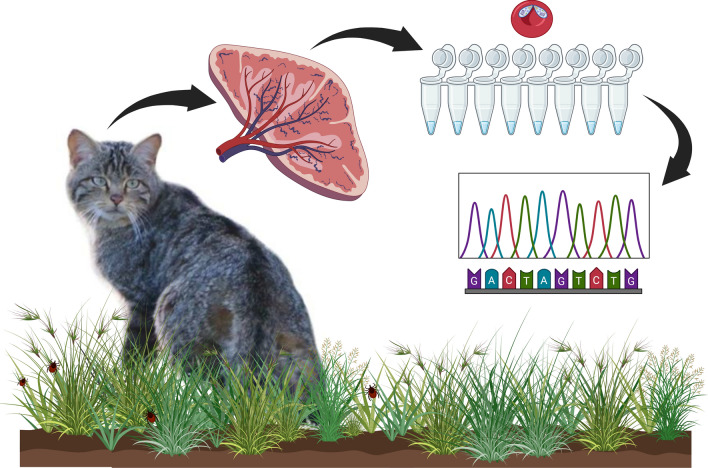

## Background

The resilience of ecosystems is increasingly threatened by the diminishing separation between domestic and wild mammals [1]. This convergence poses significant risks for the transmission of diseases between wildlife and domestic carnivores, thus possibly facilitating spill over events and the emergence or re-emergence of zoonoses. The European wildcat (*Felis silvestris*) and the domestic cat (*F*. *catus*), being closely related to each other [2], may exemplify this concern.

The European wildcat has a broad but fragmented geographical range across Western-Central Europe, the Apennine Peninsula, the Iberian Peninsula, Eastern-Central, Eastern, and South-eastern Europe [3]. European wildcat populations in Germany significantly declined owing to human persecution until the early 20th century. During this period, wildcats were restricted to refugia in the low mountain regions, including the Palatinate Forest, Eifel, and Harz Mountains [4]. However, recent monitoring indicates a strong recovery in various regions far from these origins, including the Bavarian Forest in the southeast and the Lueneburg Heath in the north of Germany [5]. The current estimate is that there are between 7000 and 10,000 wildcats living in Germany [6]. Domestic cats have been present in Europe since their introduction by the Romans [7, 8], and their numbers continue to rise [9]. Recently, the domestic cat population in Germany has nearly doubled to over 15 million [10], outnumbering wildcats by at least 1000 to 1. The increasing populations of both species likely lead to more direct and indirect (through arthropod vectors) contact, despite domestic cats being more common near human settlements and less frequent in preferred wildcat habitats. Furthermore, habitat disturbance, such as forest fragmentation or urbanization of rural areas, may also facilitate the presence of domestic cats in wildcat habitats, thereby further enhancing the likelihood of encounters between the two species [9]. From a reservoir host perspective, the overlapping habitats of wildcats and domestic cats, represented by feral cats in sylvatic areas, may expand the ecological niche for zoonotic pathogens [11]. In Europe, domestic cats are known reservoirs for zoonotic pathogens, such as *Toxoplasma gondii* and *Bartonella henselae* [12, 13]. Furthermore, they are known to be hosts for hard ticks [14], which are vectors for zoonotic pathogens, such as *Rickettsia* spp., the obligate intracellular bacteria causing tick-borne rickettsiosis in humans [15]. While it is known that domestic cats are reservoirs for the cat flea (*Ctenocephalides felis*), which is the vector of the causative agent of *R*. *felis*, little is known about wildcats [16, 17] with a lack of evidence of reservoir function for *Rickettsia* spp. so far [18]. Other zoonotic, tick-borne bacteria from the order Rickettsiales, such as *Anaplasma phagocytophilum* and *Neoehrlichia mikurensis*, are found in domestic and wild mammals [19, 20]. *Anaplasma phagocytophilum* causes granulocytic anaplasmosis in horses, dogs, and humans. Clinical cases of anaplasmosis are described in domestic cats all over Europe, however, only in small numbers [21]. *Neoehrlichia mikurensis* causes unspecific symptoms mostly in immunosuppressed patients and animals [22]. The status of wildcats in the transmission cycle of both pathogens is still unclear. *Bartonella* spp. are vector-borne zoonotic bacteria with a broad range of hosts, vectors, and clinical symptoms. For instance, domestic cats are the main reservoirs for *B*. *henselae*, the causative agent of cat scratch disease in humans [13]. The reservoir function of wildcats remains under debate. Cytauxzoonosis, a tick-borne disease affecting domestic and wild felids, is caused by apicomplexan haemoparasites of the genus *Cytauxzoon*. *Cytauxzoon felis*, which is primarily present in North America, is the most well-known species, provoking severe, often fatal symptoms in domestic cats. In North America, the natural reservoir of *C*. *felis* is the bobcat (*Lynx rufus*) [23]. Recently, molecularly distinct *Cytauxzoon* spp. have been reported in domestic cats with symptomatic and fatal infections from various European countries, including Italy, France, and Germany [24–26]. Despite the significance of cytauxzoonosis in domestic cats, the disease is understudied in Europe. It has been postulated that both the Eurasian lynx (*L*. *lynx*) and the Iberian lynx (*L*. *pardinus*) may act as asymptomatic reservoirs, analogous to the bobcat in the USA [27, 28]. The European wildcat likely plays a role in *Cytauxzoon* spp. transmission, but data on its reservoir potential remain scarce [18]. An arthropod vector analogous to *Amblyomma americanum* and *Dermacentor variabilis* in Northern America has yet to be identified in Europe. Additional research is required to elucidate the roles of domestic cats and potential wildlife hosts in the biology, ecology, epidemiology, and clinical manifestations of feline cytauxzoonosis in Europe to formulate effective disease mitigation strategies [28]. The reciprocal ecological interactions between domestic cats (including feral and stray cats) and wildcats regarding vector-borne pathogens (VBPs) and their roles as hosts remain poorly understood. Knowledge regarding VBPs in wildcat populations is limited and fragmented, and their potential implications for domestic cat populations and vice versa are not known. Thus, the aims of the study were to (1) collect wildcat samples from a stable wildcat population to (2) detect VBPs, such as *N*. *mikurensis*, *A*. *phagocytophilum*, *Rickettsia* spp., *Bartonella* spp. and Piroplasmida, such as *Cytauxzoon* spp.

## Methods

### Study area and sample collection

Wildcat specimens for this study were obtained between 2018 and 2020 from the federal state of Rhineland-Palatinate, southwestern Germany either as roadkill or found deceased as part of the project “Monitoring of dead wildcats in Rhineland-Palatinate (Totfundmonitoring Wildkatze in Rheinland-Pfalz)” of the Rhineland-Palatinate chapter of Friends of the Earth Germany (Bund fuer Umwelt und Naturschutz Deutschland (BUND), Landesverband Rheinland-Pfalz)[29]. All individuals were investigated as part of a federal carcass-monitoring program, thus obviating the need for ethical approval. Carcasses were frozen at −20 °C and investigated at the Clinic for Birds, Reptiles, Amphibians, and Fish at Justus Liebig University (Giessen, Germany). During necropsies, species (European wildcat, domestic cat or suspected hybrid), sex and age class (juvenile, subadult, adult) of each cat were identified by morphological examination according to standardized protocols [30]. Data on the morphometric species determination and on a confirmatory genetic species analysis has been conducted and published before [29, 31, 32]. For this study, spleen and skin samples were collected from individuals belonging to *F*. *silvestris* (wildcat), only, and preserved at −20 °C until further processing.

### Sample preparation, DNA extraction for pathogen analysis

Spleen samples with an average size of 1 cm^3^ were taken and individually stored in tubes with 0.6 g of sterile ceramic beads (diameter 1.4 mm, Bertin Technologies, Montigny-le-Bretonneux, France) to which 600 µL phosphate-buffered saline (PBS) were added. The skin samples were likewise individually processed, however, with 0.6 g of sterile steel beads (diameter 2.8 mm, Bertin Technologies, Montigny-le-Bretonneux, France) instead of ceramic beads. Thereafter, all samples were homogenized in the Precellys^®^24 tissue homogenizer (Bertin Technologies, Montigny-le-Bretonneux, France) at 5000 rpm for 2 × 30 s with a 15 s break in between for all samples. A second homogenization step under the same conditions was repeated for skin samples only. DNA from all samples was extracted individually using the QIAamp DNA Mini Kit® (Qiagen, Hilden, Germany) following the manufacturer’s instructions. To exclude contamination during each DNA extraction run, a DNA isolation control was added with PBS instead of sample material. DNA quality and quantity were determined with a spectrophotometer (NanoDrop^®^ 2000c, Peqlab Biotechnologie, Erlangen, Germany) for each sample. All DNA samples were stored at −20 °C until further examination.

### PCR methods for the detection of vector-borne pathogens

For conventional PCR regarding *Rickettsia* spp. and *Bartonella* spp. samples with higher DNA amounts were diluted to have a final DNA amount of 20–100 ng/µl per sample. Skin samples were tested for *Rickettsia* spp. and spleen samples were screened for *A*. *phagocytophilum*, *N*. *mikurensis*, *Bartonella* spp., and Piroplasmida. Real-time PCRs (qPCR) were performed in the Mx3000P Real-Time Cycler (Stratagene, Agilent Technologies Deutschland GmbH, Waldbronn, Germany). The following protocols were used, for *A*. *phagocytophilum*, the *msp2* gene with a product size of 77 bp [33], for *N*. *mikurensis*, the partial *groEL* gene (99 bp) [34], and for *Rickettsia* spp., the *gltA* gene (70 bp) [35]. Samples positive for *Rickettsia* spp. in qPCR yielding a CT value ≤ 37 were further examined to obtain the *Rickettsia* species level via sequencing (as described below) by conventional PCR targeting the *ompB* gene (811 bp) [36].

The presence of *Bartonella* spp. was analyzed by conventional PCR targeting the NADH dehydrogenase subunit (*nuoG*) with an amplicon size of 346 bp. Additionally, all samples were further analyzed in two PCRs targeting the *gltA* gene (378 bp) and a fragment of the 16S-23S rRNA ITS region (453–780 bp) [37–40].

The PCR analyses were adjusted as described in previously published PCR protocols by our group [41]. Further, spleen DNA was screened for Piroplasmida by the use of a conventional PCR targeting the 18S rRNA gene (411–452 bp) [42]. The PCR method was carried out as mentioned before [43]. Positive samples were further analyzed for the larger fragment of 18S rRNA gene [44, 45] (1335 bp) targeting *Cytauxzoon* spp. and additionally for the mitochondrial cytochrome b (*cytB*) gene using nested PCR assays as previously published (1333 bp) [46].

Negative controls with nuclease-free distilled water, in the absence of template DNA, as well as positive controls were included for each PCR reaction. Positive controls derived from *R*. *raoultii*, *B*. *henselae*, and *A*. *phagocytophilum* directly from culture and from a field strain of *N*. *mikurensis* from a positive bank vole in Germany and *Babesia* *caballi* from a positive horse.

Sequencing of PCR products for *Rickettsia* spp., *Cytauxzoon* spp. and *Bartonella* spp. was performed commercially by Eurofins Genomics Germany GmbH (Ebersberg, Germany) with the corresponding forward and reverse primers of each gene used for PCR amplification. The sequences were analyzed to species level with BioNumerics Software Ver. 7.6.3 (AppliedMaths NV, Sint-Martens-Latem, Belgium). Subsequently, a comparison was conducted to sequences present in GenBank on the Basic Local Alignment Tool (BLAST; https://blast.ncbi.nlm.nih.gov/Blast.cgi, accessed on 29 May 2024). Obtained sequences for *C*. *europaeus* were uploaded to GenBank under following accession numbers: PP882682-PP882704 [for 18S ribosomal RNA (rRNA)] and PP919607-PP919629 (for *cytB*).

### Statistical analysis

The 95% confidence intervals (95% CIs) of prevalence rates for each pathogen in examined cats were determined by means of the Clopper–Pearson method, using the Graph Pad Software (Graph Pad Software Inc., San Diego, CA, USA). The chi-square test was used to compare *Cytauxzoon* spp. prevalence between age groups. Fisher’s exact test was used with a type I error α of 0.05 to test the independence of compared prevalence rates.

## Results

### Cat sample collection

In total, samples from 117 European wildcats were included to this study (Fig. 1). The majority of European wildcats were males (*n* = 62; 53%), with females comprising 42.7% (*n* = 50). Most of the animals were adults (*n* = 75; 64.1%), followed by subadults (*n* = 26; 22.2%) and juveniles (*n* = 14; 12%). Due to insufficient carcass conditions, sex determination was not possible for five individuals (4.3%), and age determination for two individuals (1.7%) (Table 1).

**﻿Fig. 1:**
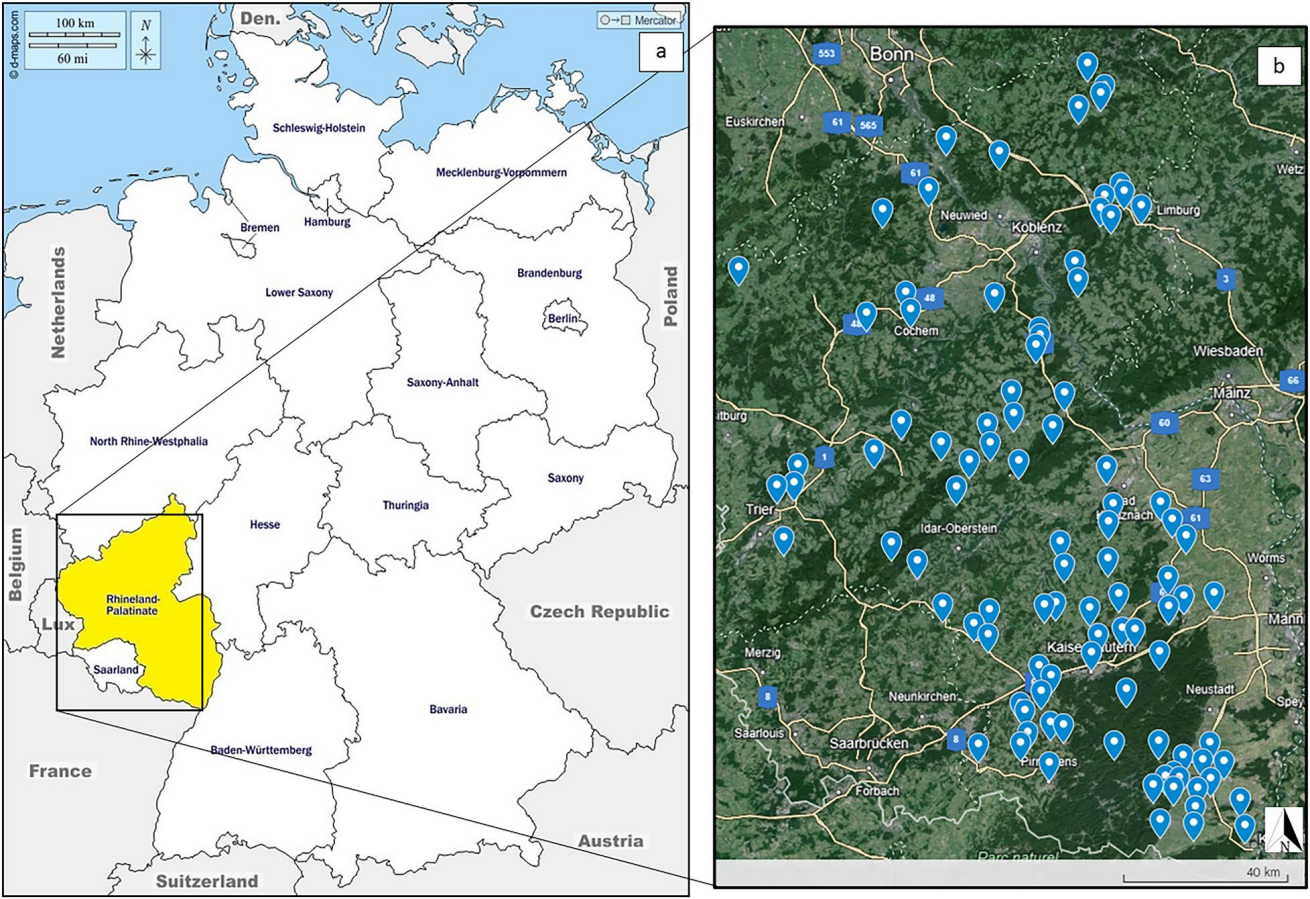
Study sites. **a** Overview of the studied area (yellow) in Germany, Rhineland-Palatinate state (https://d-maps.com/carte.php?num_car=4692 with own modifications); **b** collection points of European wildcat carcasses (blue pins) (Google Earth with own modifications)

**Table 1:** Number of European wildcats (*Felis silvestris*) per age and sex collected in Rhineland-Palatinate, southwest Germany

Age	Number of collected individuals [*n*, (%)]			
	Total	Females	Males	n.d
Juvenile	14 (12%)	3 (2.5%)	10 (8.6%)	1 (0.8%)
Subadult	26 (22.2%)	12 (10.3%)	14 (12%)	0
Adult	75 (64.1%)	35 (29.9%)	37 (31.6%)	3 (2.6%)
n.d	2 (1.7%)	0	1 (0.8%)	1 (0.8%)
Total	117 (100%)	50 (42.7%)	62 (53%)	5 (4.3%)

*n* number; *n.d.* not determined

### Prevalence and sequence analyses of vector-borne pathogens

The overall prevalence for at least one vector-borne pathogen was 75.2% in European wildcats (*n* = 117) (*n* = 88; 95% CI 66.38–82.73). The most prevalent genus found was *Cytauxzoon* with 71.8% (*n* = 84; 95% CI 62.73–79.72), followed by *Rickettsia* (*n* = 8; 6.8%; 95% CI 3–13.03), *A*. *phagocytophilum* (*n* = 3; 2.6%; 95% CI 0.53–7.31), and *Bartonella* (*n* = 1; 0.8%; 95% CI 0.02–4.67) (Table 2). None of the individuals tested positive for *N*. *mikurensis*.

**Table 2:** Prevalence of pathogens detected in 117 European wildcat (*Felis silvestris*) individuals collected in Rhineland-Palatinate, southwest Germany

Pathogen	Number of individuals positive	Prevalence in %; 95%CI
*Cytauxzoon* spp.	84	71.8%; 62.73–79.72
*Rickettsia* spp.	8	6.8%; 3–13.03
*Bartonella* spp.	1	0.8%; 0.02–4.67
*Anaplasma phagocytophilum*	3	2.6%; 0.53–7.31
*Neoehrlichia mikurensis*	0	–

*CI* confidence interval

Overall, eight wildcat individuals were coinfected with two pathogens (Table 3). The most prevalent coinfection was *Rickettsia* spp. & *Cytauxzoon* spp. (*n* = 4) and *A*. *phagocytophilum* & *Cytauxzoon* spp. (*n* = 2).

**Table 3:** Number of detected coinfections in tested European wildcats (*Felis silvestris*)

Pathogen combinations		Wildcat	Total
*Rickettsia* spp. + *Cytauxzoon* spp.	*Rickettsia* spp. + *C. europaues*	1	4
	*Rickettsia* spp. + *Cytauxzoon* spp.	2	
	*R*. *helvetica* + *Cytauxzoon* spp.	1	
*Rickettsia* spp. + *Anaplasma* spp.	*R*. *helvetica* + *A*. *phagocytophilum*	1	1
*Anaplasma* spp. + *Cytauxzoon* spp.	*A*. *phagocytophilum* + *Cytauxzoon* spp.	1	2
	*A*. *phagocytophilum* + *C*. *europaeus*	1	
*Bartonella* spp. + *Cytauxzoon* spp.	*B*. *taylorii* + *Cytauxzoon* spp.	1	1

Comparing female and male wildcats, there was no statistical difference in the prevalence of *Cytauxzoon* spp. (*P* = 1). Likewise, there was no statistical difference in the *Cytauxzoon* spp. prevalence between wildcat age groups ($\chi$ ^2^ = 1.982; *P* = 0.371; *df* = 2) (Table 4).

**Table 4:** Prevalence of *Cytauxzoon* spp. in 117 European wildcats (*Felis silvestris*) per sex and age collected in Rhineland-Palatinate, southwest Germany

Sex	Prevalence of *Cytauxzoon* spp. [*n* positive/*n* tested, (%; 95%CI)]				
	Juvenile	Subadult	Adult	n.d	Total
Male	8/10 (80%; 44.39–97.48)	10/14 (71.4%; 41.9–91.61)	27/37 (73%; 55.88–86.21)	0/1	45/62 (72.6%; 59.77–83.15)^2^
Female	2/3 (66.7%; 9.43–99.16)	6/12 (50%; 21.09–78.91)	28/35(80%; 63.06–91.56)	0/0	36/50 (70.6%; 57.51–83.77)^2^
n.d	1/1 (100%; 2.5–100)	0/0	1/3 (33.3%; 0.8–90.57)	1/1 (100%; 2.5–100)	3/5 (60%; 14.66–94.73)
Total	11/14(78.6%; 49.2–95.34)^1^	16/26 (61.5%; 40.57–79.77)^1^	56/75 (74.7%; 63.3–84.01)^1^	1/2(50%; 1.26–98.74)	84/117(71.8%; 62.73–79.72)

*n* number; *n.d.* not determined; *CI* confidence interval

^1^No statistical difference between age groups ($\chi$^2^ = 1.982; *P* = 0.371; *df* = 2)

^2^No statistical difference between sexes (*P* = 1)

Regarding sequence analyses of pathogens detected in wildcats, three out of eight samples positive for *Rickettsia* spp. qPCR were further processed through conventional PCR for sequencing (*ompB*), which revealed *R*. *helvetica*. The samples were 99.88% similar to those detected in *Ixodes ricinus* (GenBank Acc. No. MF163037) and *I*. *persulcatus* from Novosibirsk in Russia (GenBank Acc. No. Ku310591). Sequencing (*nuoG* and ITS) of the *Bartonella*-positive sample uncovered the presence of *B*. *taylorii*, which was 100% identical with a sample from *Microtus* sp. from France (GenBank Acc. No. CP083444) and an isolate from *Apodemus sylvaticus* from the UK (GenBank Acc. No. CP083693). Out of 84 samples positive for *Cytauxzoon* spp., 23 were randomly chosen for sequence analysis. Our 18S rRNA samples revealed sequences (GenBank Acc. No. PP882682-PP882704) of *C*. *europaeus*. All samples showed very high identity levels (99.84–100%) to *C*. *europaeus* from other *F*. *silvestris* samples from Germany—Saxony-Anhalt (GenBank Acc. No. ON380477) and Thuringia (GenBank Acc. No. ON380472)—as well as to *C*. *europaeus* haplogroup “major EU1” from European wildcats from Luxembourg (GenBank Acc. No. MT904044), Germany (GenBank Acc. No. MT904041), Italy (GenBank Acc. No. MT904034), Bosnia and Herzegovina (GenBank Acc. No. MT904025), and *L*. *lynx* from Romania (GenBank Acc. No.MT904027). Regarding the *cytochrome B* gene, all 23 sequenced samples (GenBank Acc. No. PP919607-PP919629) unveiled high identity levels (99.43–99.75%) likewise to *C*. *europaeus* samples from European wildcats from Germany, Hesse (GenBank Acc. No. ON856002), Thuringia (GenBank Acc. No. ON855999), and Lower Saxony (GenBank Acc. No. ON856004). All other samples were considered “*Cytauxzoon* spp.-positive” and not processed further.

## Discussion

In this study, a representative sample of European wildcats, collected from a restricted geographical area over a limited period of time, were examined for VBPs. The sampling region is known for its stable wildcat population.

Cytauxzoonosis, a vector-borne disease in domestic cats, remains debated as an emerging concern in Europe. While *C*. *felis* involves the bobcat as the primary sylvatic reservoir and ticks (*A*. *americanum* and *D*.*variabilis*) as the main vectors in the USA [47, 48], the biological life cycle of *C. europaeus* in Europe is not well understood. Eurasian lynx and Iberian lynx are considered primary reservoirs for *C*. *europaeus* [44]; however, domestic cats, both those surviving infection and subclinically infected individuals, may also act as reservoirs [49, 50]. In addition, *C*. *europeus* has been detected in wildcats from Italy, Germany, Romania, Czech Republic, Luxembourg, and Switzerland with varying prevalence rates between 19 and 69% (Table 5), suggesting wildcats as ideal reservoirs for this pathogen [18, 25, 27, 46, 51, 52]. Our study corroborates this hypothesis by reporting the highest prevalence of *C*. *europaeus* in wildcats from Europe so far. Owing to the absence of a known vector in Europe, horizontal and vertical transmission in wildcats cannot be ruled out, which was suggested in a past study on domestic kittens from one litter that tested positive [51, 53, 54]. However, it is important to note that the kittens were also tick-infested. The wildcats from our study were infested by roughly 80% with ticks of the species *I*. *ricinus*, which has been suggested as a vector [46], and more rarely with *I*. *canisuga* and *I*. *hexagonus* (Bisterfeld et al., submitted). However, neither *I*. *ricinus* nor any other tick species from Central Europe has tested positive for *Cytauxzoon* spp. to date [55, 56]. The prevalence was similarly high across all wildcat age classes, supporting the hypothesis that *C*. *europaeus* might be vertically transmitted in wildcat populations [51]*.* Future research should aim to fully understand the life cycle of *C*. *europaeus*, including testing ticks from wildcats for this piroplasm, which is planned for upcoming studies.

**Table 5:** Prevalence rates of *Cytauxzoon* spp. in European wildcats detected in this study and other European countries

Country	Detected prevalence		References
	*n* positive/*n* tested	Prevalence (95% CI)	
Italy	4/21	19% (5.45–41.91)	[51]
Italy	4/19	21% (6.05–45.57)	[52]
Romania	9/31	29% (14.22–48.04)	[46]
Switzerland	10/34	29% (15.1–47.48)	[27]
Czech Republic	5/11	45% (16.75–76.62)	[46]
Germany, Central	45/96	47% (36.62–57.34)	[18]
Germany	30/46	65% (49.75–78.65)	[46]
Luxembourg	9/13	69% (38.59–90.91)	[46]
Germany, Southwest	84/117	72% (62.73–79.72)	Present study

*n* number

Besides the high prevalence of *C*. *europaeus* in European wildcats, this species exhibits a broader distribution and higher population density compared with other suspected wildlife hosts, such as *L*. *lynx* and *L*. *pardinus*. In addition, wildcats have a significantly higher likelihood of direct contact with infected and non-infected domestic cats, making them more impactful in the transmission of *Cytauxzoon* spp. than other wild felids. This study reports the first detection of *Rickettsia* spp., specifically *R*. *helvetica*, in wildcats in Central Europe. Previously, *R*. *helvetica*, *R*. *massiliae*, and *R*. *monacensis* were identified in ticks associated with the Iberian lynx in Europe [59], and an earlier study indicated an absence of *Rickettsia* spp. in wildcats in Germany [18]. *Rickettsia helvetica*, part of the spotted fever group, is the most commonly found *Rickettsia* species in Germany and is known to cause fever, rash, and myalgia. Domestic cats are not typically considered reservoirs. Furthermore, this pathogen is more often associated with sylvatic rather than urban settings. Potential reservoir hosts include roe deer (*Capreolus capreolus*) and wild boar (*Sus scrofa*), but rodents, such as voles and mice (e.g., *Clethrionomys glareolus*, *Microtus arvalis*, *Apodemus flavicollis*), are the most commonly considered reservoirs [60–62]. In our study, eight wildcats tested positive for *Rickettsia* spp. This suggests that wildcats possibly serve as suitable hosts or may contract the pathogen temporarily through predation on infected rodents or tick bites. Further investigations into the infection routes in wildcats are needed to determine a potential reservoir function.

Similarly, one rodent-associated *Bartonella* species, *B*. *taylorii*, was detected in one wildcat in the present study. A previous study on wildcats in Germany reported that 3% of wildcats were positive for *Bartonella* spp., all of which were also rodent-associated [18]. Although domestic cats are the primary reservoirs for *B*. *henselae*, this species has not been found in wildcats [13]. These findings suggest that wildcats might be accidental hosts for rodent-associated *Bartonella* spp., possibly owing to predation [63] rather than being involved in the urban life cycle of zoonotic *B*. *henselae*. The detected *B*. *taylorii* is of unknown zoonotic potential, usually associated with shrews [64–66], being a common prey for wildcats in Germany [67].

*Anaplasma phagocytophilum* can cause severe clinical signs in humans and domestic animals. On the basis of genetic differences in the *groEL* gene, four ecotypes of *A*. *phagocytophilum* have been proposed [68]. Ecotype 1, primarily associated with clinical cases in humans and domestic animals, has been found in many host species, including humans and livestock. Ecotypes 2, 3, and 4 are mainly found in roe deer, rodents, and birds, respectively, and are less relevant for zoonotic pathogenicity [68]. The presence of *A*. *phagocytophilum* has been reported in symptomatic domestic cats in Europe [21] and in wild felids from Hungary and Romania [69, 70]. To date, wildcats from Central Europe have not been found to be positive [18]. In our study, three wildcats tested positive, but ecotyping was not conducted. However, previous research has demonstrated the presence of the highly pathogenic and zoonotic ecotype 1 in wildcats from Hungary [69].

*Neoehrlichia mikurensis* is mainly transmitted by *I*. *ricinus* ticks in Europe [19]. It is known to cause unspecific symptoms, such as fever and myalgia, mainly in immunosuppressed humans but also in dogs [19] and has been identified in several wildlife species, including carnivores, such as badgers (*Meles meles*) and brown bears (*Ursus arctos*), in Central Europe [57]. While the primary wildlife reservoir is still under debate, several rodent species are suspected to be the main reservoir [58]. *Neoehrlichia* spp. were investigated in wildcats, however, without a positive outcome. To the authors’ knowledge, there have been no prior investigations in wildcats, specifically focusing on *N*. *mikurensis*, which was absent in our study.

Apart from *Cytauxzoon* spp., the overall prevalence rate of VBPs in the examined wildcats was relatively low. The detected pathogen composition in wildcats from our study is naturally more prevalent in sylvatic mammals, which indicates a predominantly distinct wildcat population without an intermix with urban domestic cats.

## Conclusions

The low prevalence of pathogens analyzed, with the exception of *C*. *europaeus*, suggests that wildcats are more likely to act as incidental hosts than primary reservoirs for most VBPs. The absence of *N*. *mikurensis* suggests that the reservoir function of wildcats is limited for this pathogen. The lack of *B*. *henselae* and *R*. *felis* also suggests minimal interaction with domestic cats, supporting a sylvatic pathogen life cycle in wildcats. This is further evidenced by the presence of sylvatic and rodent-associated *B*. *taylorii* and *R*. *helvetica* in wildcats from our study. Although *A*. *phagocytophilum* was detected in wildcats, and a sylvatic ecotype of this pathogen appears plausible, further ecotyping is required to allocate the origin of the strains. The high prevalence of *C*. *europaeus* indicates that wildcats may serve as reservoir hosts for this piroplasm in Germany and adds to the understanding of the ecology of this understudied parasite. The results may support the assumption that the main transmission may occur through vertical transmission rather than vector-borne. The composition of the VBPs found may serve as indicators of the distinct coexistence of domestic and wildcats, as evidenced by the rather strict separation of pathogens harbored by each host population.

## Data Availability

The data supporting the findings of this study are available within the article.
